# Psychobiological risk factors for insomnia and depressed mood among hospital female nurses working shifts

**DOI:** 10.3389/frsle.2023.1206101

**Published:** 2023-11-06

**Authors:** Kochav Bennaroch, Tamar Shochat

**Affiliations:** ^1^Rambam Health Care Campus, Nursing Administration, Haifa, Israel; ^2^Cheryl Spencer Department of Nursing, University of Haifa, Haifa, Israel

**Keywords:** insomnia, depressed mood, psychobiological risk factors, hospital nurses, shift work, day work, psychobiological model, SEM analysis

## Abstract

**Introduction:**

Despite a vast body of knowledge on the associations between insomnia and depression, and although women and shift workers are at high risk for each of these conditions separately, common psychobiological risk factors for developing insomnia and depressed mood concomitantly in high-functioning shift-working female nurses have yet to be investigated within a comprehensive framework. This study examines the contribution of shift work (disruption of circadian rhythms), stress, analytical rumination, and morningness-eveningness on the development of insomnia and depressed mood among female hospital nurses.

**Objectives:**

We sought to assess the severity and prevalence of insomnia symptoms and depressed mood among hospital shift-working compared with day-working nurses; to examine associations between psychobiological risk factors with insomnia and depressed mood; and to develop a conceptual psychobiological model to describe their co-occurrence among hospital nurses.

**Methods:**

Using a cross-sectional design, we recruited female hospital nurses, shift workers (SW) and day workers (DW: only morning shifts), and assessed them for insomnia, depressed mood, stress, analytical rumination, and morningness-eveningness through validated self-administered questionnaires delivered online. Using structural equation modeling (SEM), we assessed common pathways between psychobiological factors affecting insomnia and depressed mood.

**Results:**

448 nurses completed electronic questionnaires. SW nurses (*n* = 358) compared with DW nurses (*n* = 90) had significantly higher rates of insomnia and depressed mood. SW nurses also reported significantly higher severity of insomnia, depressed mood, stress, and a tendency to eveningness compared with DW nurses. A positive linear relationship was found between insomnia and depressed mood in both SW and DW nurses. SEM showed that shift work contributed directly to insomnia and indirectly to depressed mood. The overall model showed a good fit between the empirical and the conceptual psychobiological model proposed in the study [χ_(1)_ = 0.16, *p* = 0.69, CFI = 0.99, RMSEA = 0.0001].

**Discussion:**

We found that SW nurses who reported high levels of stress and eveningness are at significantly greater risk for both insomnia symptoms and depressed mood. Findings provide the groundwork in creating a conceptual psychobiological model to examine the co-occurrence of insomnia and depressed mood phenomena in hospital nurses. This research is an important first step toward the development of interventions aimed at improving nurses' health, wellbeing and quality of life by preventing the mental burden associated with insomnia and depressed mood.

## 1. Introduction

Health professions workers in general, and nurses in particular, are traditionally employed in shifts because of the need to provide care 24 h a day. Shift work has consequences for health, functioning, and safety, including sleep disorders, insomnia, and high rates of depression (Caruso, 2014; Waage et al., 2014). Disturbances in sleep timing are related to disruption of the daily rhythm and cause insomnia in shift workers (American Academy of Sleep Medicine, 2005). There is evidence that stress is a factor associated with the negative consequences of shift work for nurse health (Clissold et al., 2002; Koh et al., 2014).

The evidence-based body of knowledge that the relationship between psychiatric illness and sleep involves bidirectional causation is growing (An et al., 2016). Symptoms of depression are major risk factors for insomnia, and depression is considered a comorbid condition among chronic insomniacs of any etiology (Luca et al., 2013). A meta-analysis of longitudinal studies supports the claim that insomnia is a significant predictor of depression (Hertenstein et al., 2019). The present cross-sectional study examined these relationships among nurses who are a population at risk of suffering from both phenomena.

Rates of insomnia and depression or depressive symptoms in hospital nurses working shifts are high. For example, one study found that 28% of nurses working shifts experienced insomnia whereas only 2% of nurses working only morning shifts did (Leyva-Vela et al., 2018). According to a survey using DSM-IV criteria (An et al., 2016), insomnia rates among hospital nurses working in shifts were about 55%. Another study of nurses from all types of hospital wards found, based on the Athens Insomnia Scale, that 50% experienced severe insomnia, and that night shift was associated with higher insomnia severity (Kousloglou et al., 2014). Survey studies also show that hospital nurses suffer from high rates of depression. For example, a study of nearly 300 hospital nurse managers using the Center for Epidemiological Study Depression (CES-D) survey questionnaire (20-item version), found a prevalence of 31% (Nourry et al., 2014). Likewise, in a study of 102 hospital nurses based on the Depression Anxiety Stress scale, the prevalence was 32% (Maharaj et al., 2018). A meta-analysis of 30 cross-sectional studies on depression among nurses in Iran showed a depression rate of 22%, higher than for the general population (5–10%; Shahri et al., 2017). Recent studies from the COVID-19 era report depression rates of 23% in a systematic review of healthcare workers (Pappa et al., 2021), and 47% in over 3,000 Chinese hospital nurses (Zheng et al., 2021).

Survey studies also assessed co-occurrence of depression and insomnia in hospital nurses. In a study of 2,000 hospital nurses in Norway, night shifts were found to increase the risk for insomnia, but not for depression (Øyane et al., 2013). Another study from the COVID-19 era in over 4,000 hospital nurses in China reported rates of 18 and 12% for moderate to severe depressive symptoms and insomnia, respectively (Liang et al., 2021). A recent survey of nearly 1,800 frontline hospital nurses during full liberalization of COVID-19 showed alarmingly high rates of both depressive symptoms (69%) and insomnia (77%, Xiao et al., 2023).

Common underlying risk factors have been reported in relation to both poor sleep and depressed mood. Studies conducted among hospital nurses working shifts in the context of stress included both biological and psychological indicators. Thus, stress has been associated with an increased prevalence of depressed mood and a decrease in positive mood, which negatively affected both cardiovascular health measures and work performance (He et al., 2014). In another study, levels of stress were associated with sleep, fatigue, satisfaction, and cardiovascular consequences (Järvelin-Pasanen et al., 2013).

Studies investigating the relationships between chronotype/morningness-eveningness, insomnia and depressed mood in shift-working (SW) nurses are inconsistent. On one hand, older nurses with an early chronotype were sleepier during the shift, whereas hypervigilance before bedtime was associated with sleepiness during the shift among nurses with a late chronotype (Zion et al., 2018). No effect of chronotype was found on psychomotor function among night nurses (Reinke et al., 2015). Vedaa et al. (2016) found that morningness predicted less symptoms of insomnia over time. In a study observing various sleep strategies employed by nurses shifting from day to night sleep, those who chose more gradual and adaptive strategies had less sleep disturbance, a later chronotype, and less cardiovascular problems (Petrov et al., 2014). The 4-year incidence of depression among nurses was 8%, with higher risk among nurses with intermediate and evening types compared to morning types (Vetter et al., 2018).

Rumination is a cognitive process with negative effects on health, including insomnia and depression (Nolen-Hoeksema et al., 1993; Perlis et al., 1997; Watkins et al., 2005). Analytical rumination, however, is conceptualized as a process in which symptoms of depression arise from continuous cognitive appraisal, resistant to distraction, over short and intense periods, and its role is to help individuals to solve challenging situations, such as those related to stressful life events (Andrews and Thomson, 2009). In the present study, we investigate the contribution of analytical rumination to depressed mood and insomnia.

Compared with the existing body of knowledge on depression and its implications for nurses, depressed mood has scarcely been studied. Depressed mood is the main symptom of depression; however, unlike the dichotomous approach to depression (yes/no), the developmental approach considers depression as a phenomenon located on the severe end of the continuum of depressed mood (Keller and Nesse, 2005). According to this approach, depressed mood is related to the collection of symptoms usually attributed to depression but is considered a normative phenomenon that develops against the background of stressful stimuli in the human environment. Few studies have examined both insomnia and depressed mood in shift working nurses (Choi et al., 2020). Here, we examined these relationships using the developmental approach.

The theoretical framework in this study is the behavioral model for the development of insomnia (Spielman et al., 1987) and the analytical rumination hypothesis for the formation of depressed mood and depression (Andrews and Thomson, 2009). According to the behavioral model, insomnia develops and worsens over time as a result of three factors: The predisposition that represents genetic or characteristic factors, that are preconditions for alertness and hyperarousal; the precipitating factor involving stressful events or life circumstances; and the perpetuating factor attributed to maladaptive behaviors, thoughts, and habits that preserve the sleep problem over time (Glovinsky and Spielman, 2006). This model is widely established to describe the pathogenesis of insomnia (Ellis et al., 2014). The analytical rumination hypothesis is an adaptive developmental theory of depression, according to which depressed mood and most cases of depression are functional and serve an attempt to adapt to stress. According to this approach, the phenomenon of rumination is an analytical process in which the person focuses on four main components of the source of stress: its cause, the aspects that require a solution, the potential solutions, and the costs and benefits of implementing each solution (Barbic et al., 2014).

Study objectives were to assess the prevalence of insomnia symptoms and depressed mood, individually and jointly, among SW hospital nurses compared with DW nurses, to examine the contribution of common factors to insomnia and depressed mood, the interrelationships between insomnia and depressed mood, and to create a conceptual psychobiological model. The definition of shift work in the current study is 8 h of work in a morning, evening, or night rotation (or another pattern of work shifts: for example, mostly evenings or mostly nights). Compared to DW nurses working only in 8 h morning shifts. *Full-time* refers to working five 8-h shifts per week.

**Figure 1:**
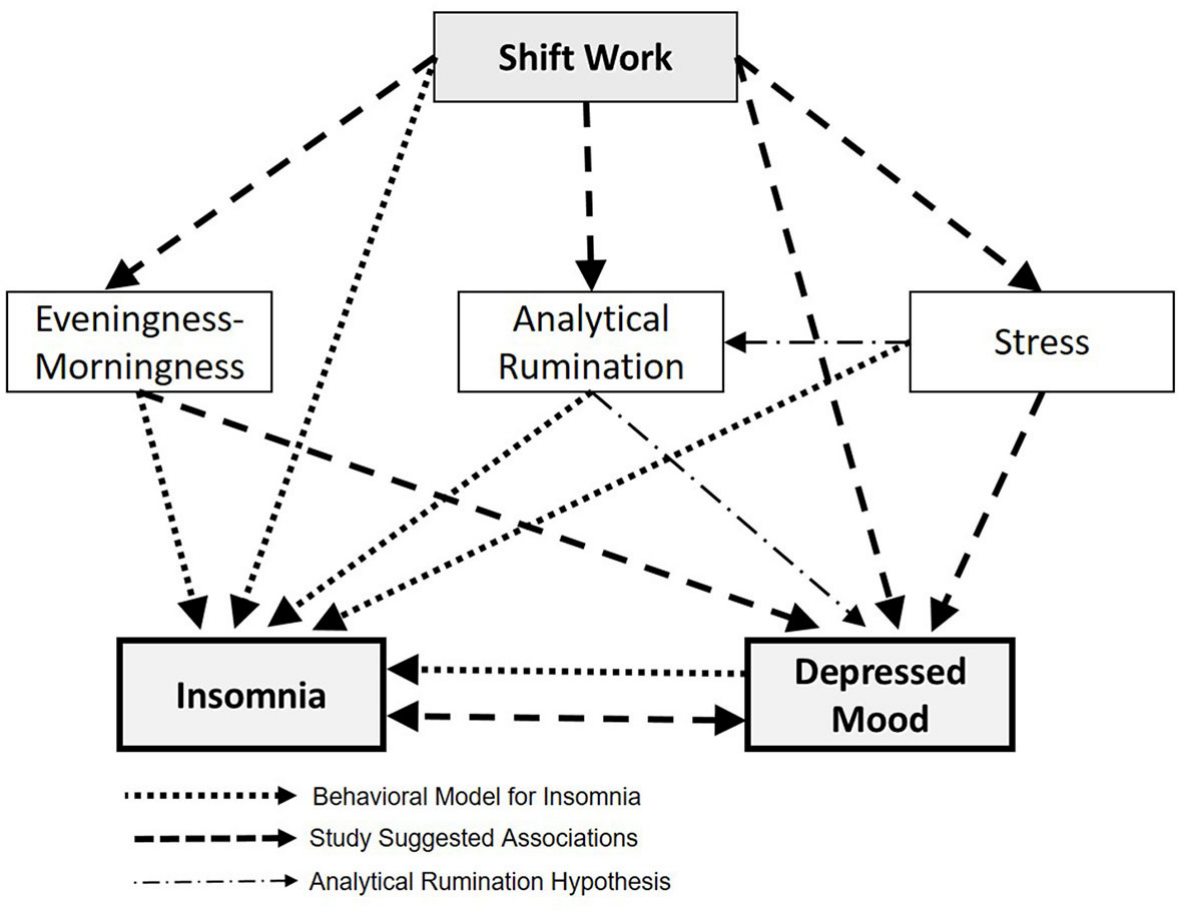
The conceptual psychobiological model for insomnia and depressed mood proposed in the study.

The research hypotheses are as follows: (1) There is a high prevalence and severity of insomnia symptoms and depressed mood, separately and jointly, among SW nurses compared with DW nurses. (2) There is a relationship between insomnia symptoms and depressed mood. Using structural equation modeling (SEM), we expect the relationship between them and their joint appearance to be explained by psychobiological risk factors (morningness-eveningness, stress, analytical rumination, shift work) that are unique and common to both phenomena—insomnia and depressed mood. Figure 1 presents our proposed conceptual psychobiological model for understanding the co-occurrence of insomnia and depressed mood among female shift working (SW) hospital nurses, who constitute a population at risk, while assessing the contribution of key potential risk factors, e.g., morningness-eveningness, that may underlie their co-occurrence.

## 2. Methods

### 2.1. Study design

A comparative cross-section study examining the presence of insomnia symptoms, depressed mood, and other psychological and biological factors among SW hospital nurses compared with DW nurses. The data was obtained between Aug 2017–Aug 2018.

### 2.2. Participants

A total of 448 Israeli hospital female nurses, all Hebrew speakers, participated in the study [333 nurses from governmental hospitals, 65 from HMO (Health Maintenance Organization) hospitals, and 50 from private hospitals]. The study group consisted of SW nurses, where the definition of shift work in the current study is 8 h of work in a morning, evening or night rotation (or another pattern of work shifts: for example, mostly evenings or mostly nights). The control group included DW nurses working only in the morning. Most of the study nurses worked full time (62%) and the rest part time. There was no age limit and no exclusion criteria. The evaluation of the sample size revealed that according to power analyzes for alpha = 0.05, a medium effect size, and a power of 0.95, the desired number of subjects is 500 subjects in total. This number includes an additional 10% for those who withdraw from the study.

Upon receiving approvals for the study from the institutional Helsinki Committee (approval #0496-16-RMB), the Rambam Hospital nursing administration, and the university's faculty ethics committee (approval number 177/18), we began recruitment. Nurses were recruited through convenience sampling by cluster, by contacting them through an email message sent to all nurses in the hospital, or through a message to designated nurses' groups on internet forums. The message offered them an opportunity to participate in the survey and explained the study aims and the research questionnaire. In addition, paper questionnaires were distributed widely to the hospital departments for those who have difficulty completing an electronic questionnaire via mobile phone/computer. Based on the ethics committees' approvals, a statement was made at the beginning of the survey, stating that completion of the questionnaire indicated participants' consent. Paper questionnaires were returned at designated mailboxes placed throughout the hospital to ensure complete anonymity.

### 2.3. Questionnaires

The study used validated self-filling questionnaires, which were translated into Hebrew and were validated. To avoid missing or incomplete entries and missing data, all questions in the electronic questionnaire were mandatory, so that participants were unable to move to subsequent items before completing previous ones. Out of 448 questionnaires returned, 51 were in paper, of which two were excluded due to at least 30% missing data. Thus, full 399 online questionnaires and 49 paper questionnaires were returned.

#### 2.3.1. Clinical demographic questionnaire

The questionnaire includes questions about demographic and personal characteristics: Age, education, number of years of education, marital status, number of children, seniority, department, shift/day work, whether the nurse holds a managerial role (deputy head nurse or higher), underlying diseases, menopause symptoms, use of drugs to induce sleep, to treat depression, pain, or chronic illness, and more.

#### 2.3.2. Insomnia severity index

The ISI (Bastien et al., 2001) is a questionnaire designed to assess sleep difficulties according to the participant's perception. It consists of seven items that assess severity of initial (sleep onset), middle (sleep maintenance), and late (early morning awakening) insomnia; degree of satisfaction with the existing sleep pattern; extent of the insomnia's interference with daily functioning; degree of prominence of the symptoms of insomnia; and feeling of distress following the difficulty of sleeping. Each item is rated on a 5-point Likert scale (0 = no problem, 4 = very serious problem). The time frame refers to the last 2 weeks. The final score ranges from 0 to 28, with higher scores indicating higher severity of insomnia, according to the following ranges: 0–7 = no clinically significant insomnia, 8–14 = mild insomnia, 15–21 = insomnia of moderate severity, 22–28 = high-severity insomnia. The questionnaire was found to be reliable and valid with a Cronbach's alpha of 0.74 (Bastien et al., 2001). Reliability and validity in the current study were established with a Cronbach's alpha of 0.87.

#### 2.3.3. The center for epidemiological studies-depression scale (CES-D 10)

The shortened version of the CED-20 (Radloff, 1977), the CES-D 10 (Kohout et al., 1993) is used to assess depressed mood and not to diagnose depression. The selection of the 10 items in this abbreviated version is based on factor research, the results of which were reported by the author of the original, longer version (Radloff, 1977). In the CES-D 10, participants are required to answer “yes” or “no” to the question “Have you felt this symptom most of the time, during the last week?” Each positive answer receives 1 point (the statements “I am happy” and “I enjoy life” receive 1 point if the answer to them is negative). Total points of 4 or more indicates depression. The CES-D 10 correlates well with other clinical rating scales and differentiates well between patients who are clinically diagnosed as experiencing depression and those who are not (Kohout et al., 1993). The reliability score decreased only slightly in the shortened version, with a Cronbach's alpha of 0.80, showing good internal consistency. The regression tests show that scores obtained from the abbreviated version can be compared with those of the original version (Kohout et al., 1993).

We used the Hebrew version of the CES-D 10, translated as part of a study on psychological and immune predictors of the development of depressive symptoms among heart patients and found to have high internal reliability, with a Cronbach's alpha of 0.87 (Gidron et al., 2001). Reliability and validity in the current study were established with a Cronbach's alpha of 0.83.

#### 2.3.4. Analytical rumination questionnaire

The ARQ (Barbic et al., 2014) is used to assess a person's analytical rumination patterns. The questionnaire enables identification of the main problem causing the person to feel stressed (the first part) and their level of rumination about the problem (the second part).

In the first qualitative part, respondents are asked to select, from a list, problems and problematic situations that are relevant to them and that caused them to feel stressed in the past 2 weeks. They are then asked to underline one problem that made them feel the worst.

The second part opens with the question “During the last 2 weeks, when you started thinking about your problems lately, to what extent did you do the following actions?” and follows with 20 items about the analytical rumination process. Example items are “I tried to find the answer to my problems” and “I tried to understand why I had these problems.” Each item is scored on a 4-point Likert scale. Answer options range from 1 (never) to 4 (all the time). The score range is 20–80; higher scores indicate a higher level of analytical rumination. The questionnaire is suitable for the population experiencing depressive symptoms and was found to be particularly effective in quantifying the level of analytical rumination upon the onset of depressive symptoms, without gender, age, or response time biases (Barbic et al., 2014). For the purposes of the present study, in the first qualitative part, the problem “difficulties at school” was replaced by “difficulty working shifts.” Reliability and validity in the current study were established with a Cronbach's alpha of 0.97.

#### 2.3.5. The composite scale of morningness

The CS/CSM (Smith et al., 1989) is a 13-item, self-answer questionnaire for determining a person's morningness-eveningness. The questionnaire is scored by summing all the answers for each item, and participants can choose one answer from four or five options. Scores range from 13 (extreme evening type) to 55 (extreme morning type): a total score of 22 or less indicates evening type, a score of 23–43 indicates intermediate type, and a score of 44 or more indicates morning type. Reliability and validity in the current study were established with a Cronbach's alpha of 0.89.

#### 2.3.6. The perceived stress scale

The PSS-10 (Cohen and Williamson, 1988) measures the level at which individuals evaluate stressful situations in their life. The questionnaire is an abbreviated version of the PSS-14 (Cohen et al., 1983) and contains 10 questions through which participants assess how unpredictable, uncontrollable, and busy their life is by indicating the frequency of thoughts and feelings in the last month, on a 5-point Likert scale, ranging from 0 (never) to 4 (very often). The questions are general and do not focus on specific events or specific experiences. The questionnaire has one summary score between 0 and 40; a low score expresses a lower stress level, and a high score expresses a higher stress level (Al Kalaldeh and Abu Shosha, 2012). In the current study, an average range of the summarizing score was determined to be between 0 and 5, where 5 expresses the highest stress level. Reliability and validity in the current study were established with a Cronbach's alpha of 0.83.

### 2.4. Data analysis

The received data were produced as an Excel file in a raw form without the possibility of identifying the participants. After receiving the data, we confirmed that the number of questionnaires met the study requirements. The classification of the population into a study group (shift work) and a control group (day work, mornings only) was done according to the answers in the demographic data questionnaire. Based on the answers in the questionnaires, we performed statistical analyses to test the research hypotheses.

First, statistical analysis was descriptive in which averages, standard deviations, and prevalence were calculated for the characteristics of the sample and the study variables, and the normality of the distribution of the main variables was tested. Second, regressions were carried out on the dependent variable **insomnia**, in which factors found to be related to insomnia were introduced (shift work, stress, morningness-eveningness, depressed mood, and analytical rumination) in order to assess their relative contribution to the appearance of insomnia among the nurses. Regressions were also run on the dependent variable **depressed mood** to assess the relative contribution of factors found to be associated with depressed mood (shift work, stress, morningness-eveningness, insomnia and analytical rumination) to the appearance of depressed mood among the nurses. Finally, the research hypotheses were examined according to the research objectives using the Structural Equation Modeling (SEM) and given to the results of path coefficients. SEM is a method of multivariate statistical analysis and a combination of factor analysis, path analysis and regression, and is a comprehensive statistical approach to identify hidden variables related to an index (Stein et al., 2017). Structural equation models are widely used in empirical research to investigate relationships among variables. We used the maximum likelihood estimation (MLE) robust extraction method for parameter estimation. The following indices were used to assess model fit: (1) Ratio of chi square and degrees of freedom: if the value of the equation was equal or smaller than two, the fit was perfect (2) Goodness of fit index (GFI), comparative fit index (CFI), and the adjusted goodness of fit index (AGFI). Recommended values greater than 0.9 were considered to represent good fit; (3) Root mean square error of approximation (RMSEA). Recommended values under 0.08 indicate perfect fit (Hair, 2010).

Using SEM, we explored the relationship among the six components, namely, shiftwork, stress, analytical rumination, eveningness-morningness, depressed mood and insomnia, examining the compatibility between the empirical model and the conceptual psychobiological model proposed. All analyses were performed using SPSS version 23 software.

## 3. Results

### 3.1. Demographic and clinical characteristics of participants

Table 1 lists categorical demographic data for the whole sample (*n* = 448) and for SW nurses (*n* = 358) and DW nurses (*n* = 90) separately. Significant differences can be seen for education (χ^2^ = 20.55, *p* < 0.001), marital status (χ^2^ = 13.43, *p* < 0.01), weekly work hours as a proportion to a 40-hour work week (χ^2^ = 18.01, *p* < 0.001), type of hospital department (χ^2^ = 85.36, *p* < 0.001), managerial role (χ^2^ = 116.18, *p* < 0.001), and use of antidepressants (χ^2^ = 7.1, *p* < 0.01). See additional demographic comparisons in Table 1.

**Table 1:** Categorical demographic variables for the overall sample and comparison between SW and DW nurses.

**Variable**	**Overall** ***N =*** **448**		**SW nurses** ***n =*** **358**		**DW nurses** ***n =*** **90**		**χ^2^**
	**Frequency**	**%**	**Frequency**	**%**	**Frequency**	**%**	
**Hospital type**							
Governmental	333	74.3	258	72.1	75	83.3	5.27
HMO	65	14.5	58	16.2	7	7.8	
Private	50	11.2	42	11.7	8	8.9	
**Education**							
RN	39	8.7	37	10.4	2	2.2	20.55^***^
BA RN	223	49.8	190	53.2	33	36.7	
MA RN	184	41.1	129	36.1	55	61.1	
PhD RN	0						
LPN	1	0.2	1	0.3	0	0	
**Family status**							
Single	77	17.2	72	20.1	5	5.6	13.43^**^
Married	330	73.7	251	70.1	79	87.8	
Single mother	36	8.0	30	8.4	6	6.7	
Widow	5	1.1	5	1.4	0	0	
**Children under 12 y**							
Yes	203	45.3	164	45.8	39	43.3	0.178
No	245	54.7	194	54.2	51	56.7	
**Weekly work hours as a proportion to a 40-h work week**							
75%	78	17.4	69	19.3	9	10.0	18.01^***^
88%	55	12.3	52	14.6	3	3.3	
100%	279	62.4	206	57.7	73	81.1	
< 75%	35	7.8	30	8.4	5	5.6	
**Departments in hospital where the nurse works**							
ICU/OR	78	17.4	68	19	10	11.1	85.36^***^
Admitting departments	240	53.6	209	58.4	31	34.4	
Outpatient clinics/nursing administration	75	16.7	31	8.7	44	48.9	
Emergency room	24	5.4	23	6.4	1	1.1	
Combination of several depts.	31	6.8	27	7.6	4	4.4	
**Managerial role**							
Yes	149	33.3	76	21.2	73	81.1	116.18^***^
No	299	66.7	282	78.8	17	18.9	
**Menopause symptoms**							
Yes	65	14.5	48	13.4	17	18.9	1.74
No	383	85.5	310	86.6	73	81.1	
**Background diseases**							
None overall	309	69	248	69.2	61	67.7	2.107
Yes overall	139	31	101	28.2	25	27.8	
Metabolic	25	5.6	21	6.3	4	4.7	
Overweight/dyslipidemia	51	11.4	37	11.2	14	16.3	
Cardiovascular	15	3.3	14	4.2	1	1.2	
Chronic pain	35	7.8	29	8.8	6	7	
Other	13	2.9					
**Daily use of analgetics**							
Yes	28	6.3	22	6.1	6	6.7	0.033
No	420	93.8	336	93.9	84	93.3	
**Sleep medication**							
Yes	67	15	56	15.6	11	12.2	0.661
No	381	85	302	84.4	79	87.8	
**Use of antidepressants**							
Yes	31	7.0	19	5.3	12	13.3	7.102^**^
No	415	93	337	94.7	78	86.7	
**Chronic disease medication**							
Yes	100	22.3	74	20.7	26	28.9	2.802
No	348	77.7	284	79.3	64	71.1	

^**^p < 0.01, ^***^p < 0.001.

No differences were found between the groups for hospital type, children under the age of 12, menopausal symptoms, background diseases, daily use of analgesics, or use of sleep medication or medication for chronic disease.

Table 2 describes the whole sample and compares SW and DW nurses for the means of continuous demographic variables. Differences emerged between the groups for age and years of seniority: DW nurses are older (t = −7.296, *p* < 0.001) and more senior (t = −6.442, *p* < 0.001) than SW nurses. In addition, differences emerged for amount of sleep hours: SW nurses slept less on working days than DW nurses did (t = −1.97, *p* < 0.05).

**Table 2:** Means and standard deviations of continuous demographic variables of the entire sample and comparison between SW and DW nurses.

**Variable**	**Overall** ***N =*** **448**				**SW nurses** ***n =*** **358**			**DW nurses** ***n =*** **90**			**t**
	**N**	**mean**	**SD**	**Range**	**N**	**Mean**	**SD**	**N**	**Mean**	**SD**	
Age	448	41.70	9.74	22–67	358	40.27	9.67	90	47.34	7.81	−7.296^***^
Number of children under 12 y living at home	448	0.84	1.09	0–5	358	0.85	1.12	90	0.79	0.989	0.469
Professional Seniority in years	448	16.58	10.79	0–44	358	15.11	10.614	90	22.46	9.43	−6.442^***^
Menopausal symptoms severity	99	3.93	2.43	1–10	74	3.97	2.42	25	3.80	2.53	0.306
Number of days absenteeism	448	0.53	2.33	0–30	358	0.53	2.43	90	0.52	1.84	0.031
Number of sleeping pills a week	448	0.45	1.35	0–7	358	0.45	1.34	90	0.44	1.40	0.051
Number of sleeping hours on work days	448	6.15	1.3	1–12	358	6.09	1.4	90	6.39	1.01	−1.97^*^
Number of sleeping hours on days off	448	8.24	1.7	1–12	358	8.29	1.9	90	8.01	1.27	1.7

^*^p < 0.05, ^***^p < 0.001.

No differences were found between hospitals for any of the study variables: insomnia, mood, stress, morningness-eveningness, or analytical rumination.

Table 3 lists means (sd) of the continuous research variables for the overall sample, comparing SW and DW nurses. Significant differences emerged between groups for average scores of insomnia symptoms (t = 4.11, *p* < 0.001), depressed mood (t = 3.48, *p* < 0.001), stress (t = 2.12, *p* < 0.05), and morningness-eveningness (t = −5.93, *p* < 0.001).

**Table 3:** Means and standard deviations of the continuous research variables, overall sample and comparing between SW and DW nurses.

**Variable**	**Overall *N =* 448**	**SW nurses *n =* 358**	**DW nurses *n =* 90**	**t**
	**M (sd)**	**M(sd)**	**M(sd)**	
Insomnia	10.85 (6.41)	11.47 (6.21)	8.41 (6.66)	4.11^***^
Depressed mood	3.36 (2.87)	3.6 (2.86)	2.43 (2.74)	3.48^***^
Stress	2.76 (0.63)	2.8 (0.63)	2.64 (0.61)	2.12^*^
Morningness-eveningness	35.48 (8.09)	34.39 (7.88)	39.84 (7.45)	−5.93^***^
Analytical rumination	47.48 (15.34)	47.6 (15.52)	46.9 (14.95)	0.4

^*^p < 0.05, ^***^p < 0.001.

### 3.2. Hypothesis 1

The **prevalence** of insomnia symptoms and depressed mood in the entire sample (*N* = 448) show that two-thirds of the nurses report mild to severe insomnia and that 43% report a depressed mood. The rate of nurses without insomnia is highest (50.2%) among nurses with normal mood, and the rate of depressed mood is highest (41.4%) among nurses with moderate insomnia (χ^2^ = 93.27, *p* < 0.001). Significant differences were found between the groups for all variables: Insomnia **severity** (*p* < 0.05), mood (*p* < 0.01), and morningness-eveningness (χ^2^ = 22.08, *p* < 0.001). Most nurses in the overall sample are intermediate types (77.5%), with rates for SW nurses higher than for DW nurses (80.2 vs. 66.7%). Among DW nurses, there is a higher rate of morning type nurses than for SW nurses (33.3 vs. 14%).

**Figure 2:**
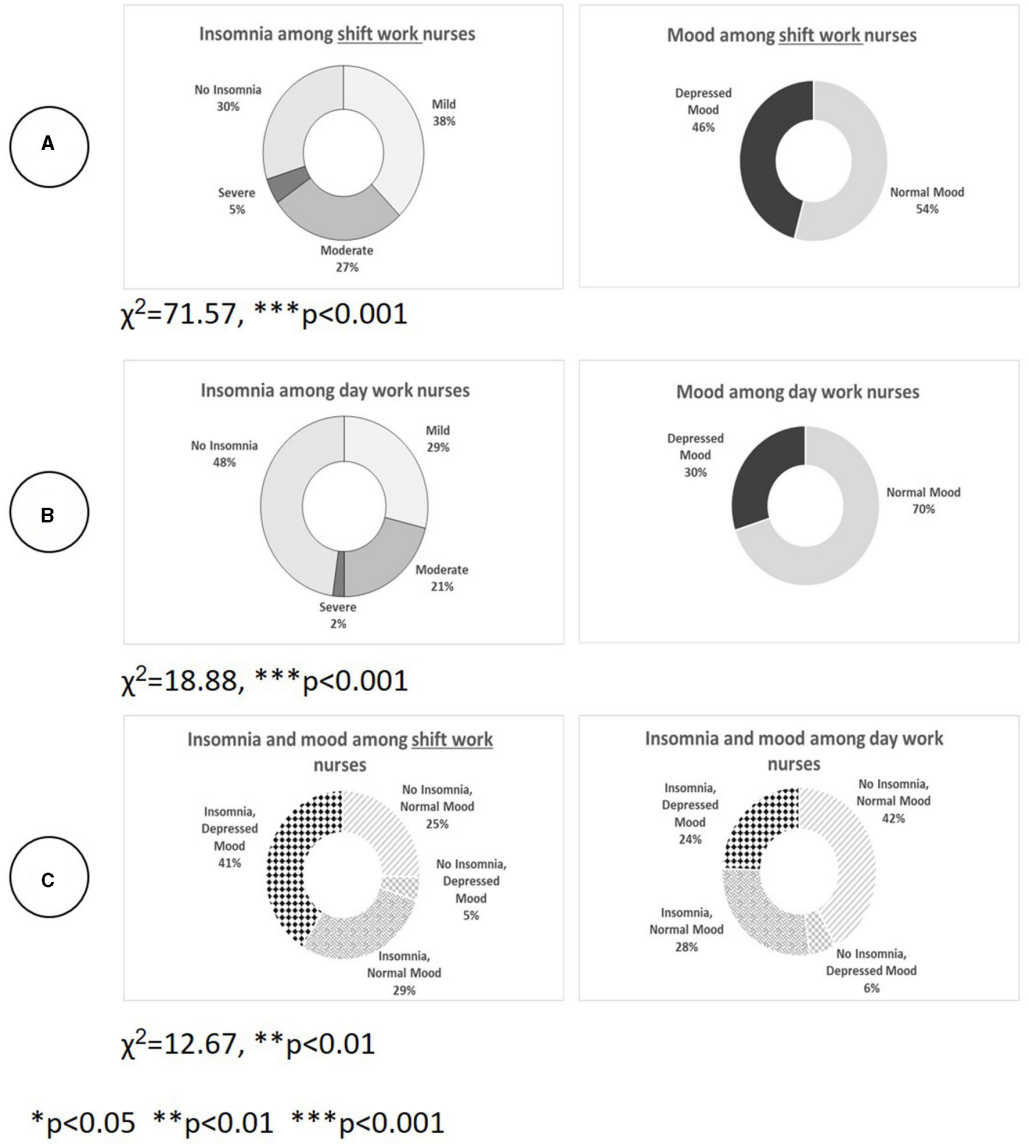
Prevalence of insomnia and depressed mood (separately and jointly) among SW nurses compared with DW nurses. **(A)** Shows that for SW nurses, the rate of mild, moderate, and severe insomnia is 70% and the rate of depressed mood is 46% (χ^2^ = 71.57, *p* < 0.001). Most SW nurses experience mild insomnia (37.7%), and a third of them, moderate-to-severe insomnia. **(B)** Shows that among DW nurses, who work only in the morning, the percentage who experience mild, moderate, or severe insomnia is 52%; that 30% have a depressed mood; and that 23% experience moderate-to-severe insomnia. The highest rate of depressed mood exists among nurses with moderate insomnia (58%, χ^2^ = 18.88, *p* < 0.001). **(C)** Shows the joint prevalence of insomnia and depressed mood for the whole sample and compares SW nurses with DW nurses. There is a significant difference between the groups. Among DW nurses, the highest rate is for the combination of no insomnia and a normal mood (42%); among SW nurses, the most common combination is insomnia with a depressed mood (41%, χ^2^ = 12.67, *p* < 0.01).

Figure 2 depicts the prevalence of the categorical dependent variables in the study, insomnia and depressed mood (separately) among SW nurses (*n* = 358, Figure 2A) compared with DW nurses (*n* = 90, Figure 2B) and jointly (Figure 2C).

A Pearson correlation test for the relationship between the two dependent variables in the study, insomnia and depressed mood, showed that their relationship is significant (Sig. = 0.01 2-tailed), positive, and strong for the whole sample (*N* = 448, r_*p*_ = 0.542), for SW nurses (*n* = 358, r_*p*_ = 0.527), and for DW nurses (*n* = 90, r_*p*_ = 0.534).

### 3.3. Hypothesis 2

Table 4 describes the Pearson correlations between the dependent and independent variables defined for the SEM model, in the overall sample (*N* = 448). All relationships were found to be significant (Sig = 0.01 2-tailed) except for the correlation between analytical rumination and shift work. All relationships between the variables are positive except for the negative relationship between morningness-eveningness and stress, mood, rumination, insomnia, and shift work. Thus, SW nurses who are evening types showed higher stress, lower mood, higher analytical rumination, and greater insomnia severity.

**Table 4:** Pearson correlations between SEM model variables in the overall sample, *N* = 448.

**Variable**	**Stress**	**Mood**	**Morningness-eveningness**	**Analytical rumination**	**Insomnia**
Mood	0.596^**^				
Morningness-eveningness	−0.175^**^	−0.278^**^			
Analytical rumination	0.345^**^	0.328^**^	−0.137^**^		
Insomnia	0.398^**^	0.542^**^	−0.272^**^	0.260^**^	
Shift work	0.100^*^	0.162^**^	−0.270^**^	0.019	0.191^**^

^*^Correlation is significant at the 0.05 level (2-tailed). ^**^Correlation is significant at the 0.01 level (2-tailed).

We conducted regression analysis to predict insomnia based on the shift-work variable alone and found that insomnia can be explained by shift work (F_(1)_ = 16.93, *p* < 0.001). Shift work explained about 4% of the variance in insomnia. To predict insomnia based on the variables shift work, mood, stress, morningness-eveningness, and analytic rumination, we ran a multiple regression analysis. The results showed that insomnia can be significantly explained by these variables (F_(5)_ = 43.3, *p* < 0.001), which accounted for 33% of the variance. The findings indicate that all the variables, with the exception of analytical rumination, significantly explain the variable insomnia.

Regression analysis, to predict depressed mood based on the shift-work variable alone, showed that depressed mood can be significantly explained by shift work (F_(1)_ = 12.08, *p* = 0.001). The predicting variable shift work explained about 3% of the variance in depressed mood. To predict depressed mood based on the variables shift work, insomnia, stress, morningness-eveningness, and analytical rumination, we ran a multiple regression analysis. The results show that depressed mood can be explained by all the predicting variables (F_(5)_ = 82.78, *p* < 0.001), which explained 48% of the variance of depressed mood.

**Figure 3:**
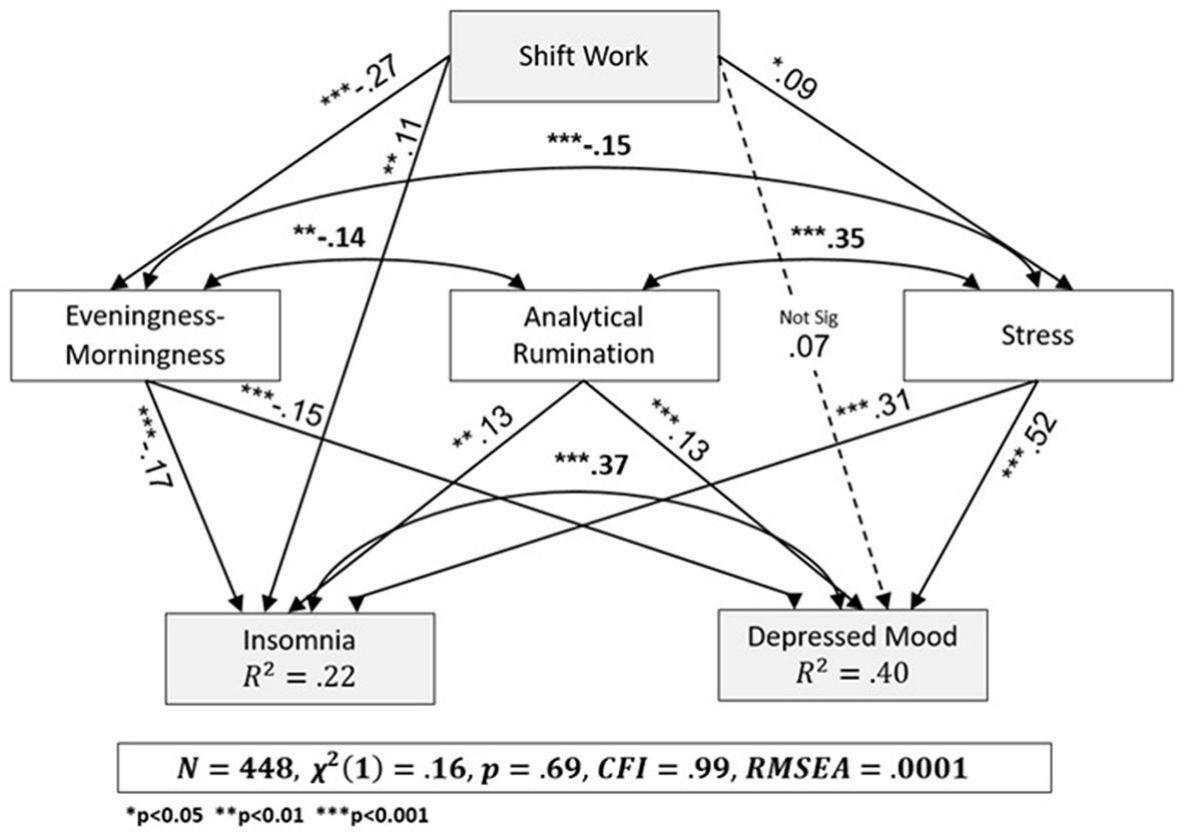
SEM analysis.

Figure 3 summarizes the SEM analysis. The model was found to have good fit indices between the empirical model and the proposed conceptual psychobiological model [χ (1) = 0.16, *p* = 0.69, CFI = 0.99, RMSEA = 0.0001].

Shift working nurses reported significantly greater eveningness relative to DW nurses (β = −0.27, *p* < 0.001). The significant positive relationship between shift work and stress (β = 0.09, *p* = 0.035) indicates that SW nurses experience higher stress. The non-significant relationship between shift work and depressed mood (β = 0.07, *p* = 0.08) and the significant positive relationship between shift work and insomnia (β = 0.11, *p* = 0.01) means that SW nurses experience more insomnia. We found a positive relationship between stress and depressed mood (β = 0.52, *p* < 0.001) and insomnia (β = 0.31, *p* < 0.001), such that higher levels of stress are combined with depressed mood and higher severity of insomnia. The significant negative relationship between morningness-eveningness and depressed mood (β = −0.15, *p* < 0.001) and insomnia (β = −0.17, *p* < 0.001) means that nurses who are evening types experience more depressed mood and insomnia. There is a significant positive relationship between analytical rumination and depressed mood (β = 0.13, *p* = 0.001) and insomnia (β = 0.13, *p* = 0.005), which indicates that a higher level of rumination is accompanied by higher levels of depressed mood and higher severity of insomnia. We found a positive significant relationship between stress and analytical rumination (β = 0.35, *p* < 0.001). We also found a significant negative reciprocal relationship between stress and eveningness (β = −0.15, *p* = 0.001), and between analytical rumination and eveningness (β = −0.14, *p* < 0.004), suggesting that evening-type nurses are prone to ruminate more. Finally, we found a significant positive reciprocal relationship between insomnia and depressed mood (β = 0.37, *p* < 0.001), indicating that the phenomena affect one another.

In conclusion, shift work contributed directly to insomnia but not to depressed mood. Analytical rumination, stress, and eveningness are associated with insomnia and depressed mood. Eveningness and stress, but not analytic rumination, mediate the relationship between shift work, insomnia, and depressed mood. The model also shows that insomnia and depressed mood are positively related to each other.

## 4. Discussion

This study examined the contribution of psychological and biological risk factors, i.e., shift work, stress, morningness-eveningness, and analytical rumination to insomnia and depressed mood among female hospital nurses in Israel, comparing shift workers with day workers. We also examined the prevalence and severity of insomnia symptoms and depressed mood, separately and jointly, among these two groups.

We found a significantly higher prevalence and severity of both insomnia symptoms and depressed mood among SW nurses; SW nurses who reported high levels of stress and eveningness were at significantly greater risk for both insomnia and depressed mood. SEM showed that shift work contributed directly to insomnia but indirectly to depressed mood. Morningness-eveningness and stress mediated the associations between shift work and both insomnia and depressed mood. Analytical rumination, stress, and eveningness were directly associated with insomnia and depressed mood. The overall model showed a good fit between the empirical and psychobiological models.

### 4.1. Differences in the prevalence and severity of insomnia symptoms and depressed mood between SW nurses and DW nurses

Hypothesis 1 predicted differences in the prevalence and severity of insomnia symptoms and depressed mood. First, we hypothesized that the prevalence of insomnia will be higher for SW nurses. This hypothesis was confirmed, and the finding is consistent with those of a study that examined rates of insomnia among hospital nurses who work shifts compared with day workers (28 vs. 2%; Leyva-Vela et al., 2018). Second, we hypothesized that SW nurses will experience more depressed mood than DW nurses. We found that the prevalence of depressed mood is higher among shift workers, a finding supported by other studies (Chiou et al., 2013; Lee et al., 2015).

We also found the co-occurrence of insomnia symptoms and depressed mood among SW nurses to be more common than among DW nurses (41 vs. 24%). Few studies examined the co-occurrence of insomnia and depression or depressed mood among hospital nurses, and these referred to different populations and shifts. In one study, the prevalence of insomnia in nurses who currently or previously worked the night shift only was associated with higher levels of insomnia than for nurses who had never worked the night shift. Furthermore, depression was not related to night-shift work experience (Øyane et al., 2013). In another study, 59% of nurses who worked alternating shifts reported poor sleep quality accompanied by more depressive symptoms. Depression, anxiety, and work atmosphere were independent predictors of sleep quality (Hsieh et al., 2011).

A possible explanation for the high rates of depressed mood is the combination of insomnia and short sleep duration (6 h), which has been found to increase the incidence of depression among adults (Fernandez-Mendoza et al., 2015). In a study of the relationship between sleep duration, mood, and stress management among healthcare workers, sleep durations of 6–7 and 5–6 h were associated with a poorer mood than sleep durations of 7 h or more (Pérez-Fuentes et al., 2019). A study of the relationship between work times and hours (shift work and morning work) and depressed mood in the general working population found that among women, working five shifts per week was associated with a higher incidence of depressed mood (Driesen et al., 2010). Although in Israel, working five shifts a week is considered full-time (40-h work week), we found no relationship between number of weekly working hours, for either SW or DW nurses, and rates of depressed mood (findings not shown). However, we demonstrate for the first time a co-prevalence of insomnia and depressed mood among hospital nurses, more among SW nurses than among DW nurses.

Furthermore, we predicted that the severity of insomnia symptoms and depressed mood would be higher for SW nurses than for DW nurses, and this hypothesis was confirmed. Support for these findings, albeit partial, can be found in a study showing that high rates of severe insomnia were related to night-shift work (Kousloglou et al., 2014), as well as in a study that associated more severe levels of insomnia with a high level of anxiety and low sleep hygiene among nurses working on a fixed-shift schedule compared with nurses working alternating shifts (Chou et al., 2015).

Support for higher severity of depressed mood among SW nurses may be found in a study showing that nurses who worked shifts were 1.5 times more likely to experience an increase in the severity of depressive symptoms than nurses who worked mornings only (Lee et al., 2015). Another study found a relationship between severity of depressed mood symptoms, type of department, and full/part time work (Chiou et al., 2013); however we did not find such associations.

### 4.2. Interrelationships between insomnia symptoms and depressed mood and the contribution of psychobiological risk factors

Hypothesis 2 predicted a two-way relationship between insomnia and depressed mood and that their relationship would be explained by the unique and shared psychobiological factors morningness-eveningness, stress, analytical rumination, and shift work. This hypothesis was confirmed.

#### 4.2.1. Eveningness and stress (but not analytical rumination) mediated the association between shift work and insomnia symptoms

Nurses who worked shifts were mostly evening types and experienced more insomnia symptoms, as shown previously (Natvik et al., 2011). Current findings are supported by a study conducted among thousands of soldiers in the US Army that found that insomnia has a significant negative relationship with morningness (Stein et al., 2018). Likewise, a longitudinal study of SW nurses showed that morning typicality predicted fewer insomnia symptoms over time (Vedaa et al., 2016). The findings however are not consistent; other studies reported no relationship between morningness-eveningness and sleep quality (De Martino et al., 2013; Lee et al., 2015). Differences between the measurement tools (sleep quality/insomnia) may partially explain the inconsistent findings.

The direct, albeit weak relationship we found between shift work and stress is supported by the literature. Stress among SW nurses was manifested in increased morbidity (Collins et al., 2005; Koh et al., 2014), greater work-family conflict (Simunić and Gregov, 2012), higher workload and multiple roles of the nurse both at work and at home (Clissold et al., 2002) compared with DW nurses.

Other studies, which used SEM analysis, showed that among night shift nurses, developing insomnia between baseline and follow-up was positively associated with languidity, i.e., the tendency to become tired/sleepy upon losing sleep (Pallesen et al., 2021). Saedpanah et al. (2022) showed that workload related to shift work was directly associated with mental health and sleep quality among 300 hospital nurses. Similarly, Yeo et al. (2022) found that shift workers had poorer sleep, in the context of mistakes at work, than non-shift workers.

Analytical rumination was not associated with shift work and did not mediate between shift work and insomnia. A possible explanation for this may be found in the work of Donahue et al. (2012), who showed that hospital nurses with a “harmonious passion” for the nursing profession experienced more feelings of recovery and wellbeing and less rumination, despite the demands, difficulties, and stress factors of their work. Harmonious passion for the profession indicates an autonomous internalization of the job requirements and recognition of its importance and was protective against emotional exhaustion (Donahue et al., 2012). Since “harmonious passion” was not measured in the current study, we can only hypothesize that it may have buffered the association between shift work and analytical rumination. The relationship between rumination and insomnia has been previously reported in the literature (Carney et al., 2013); therefore, it is important in further studies to test mediating factors that moderate the relationship between rumination and insomnia, such as mental resilience. Resilience has been found to improve nurses' ability to regulate thoughts and emotions in stressful situations (Foster et al., 2018). Nevertheless, to the best of our knowledge, our findings demonstrate, for the first time, a significant and positive (albeit weak) association between analytical rumination and insomnia symptoms.

#### 4.2.2. Eveningness and stress (but not analytical rumination) mediated the association between shift work and depressed mood

In the present study, we found an indirect association between shift work and depressed mood, mediated by eveningness and stress. A possible explanation for this indirect association may be related to the mode of shift work. According to Hall et al. (2018), nurses who worked in shifts that change quickly (e.g., morning-night-evening) or changed in an undefined manner were at the highest risk of depression. In our study, we did not make this distinction.

Eveningness mediated the association between shift work and depressed mood. Both shift work and eveningness are associated with higher levels of depressed mood because they represent a state of circadian dysnchrony (Foster et al., 2013). Circadian misalignment has been found to be an important biological component of mood vulnerability, and individuals who engage in shift work are susceptible to its deleterious effects on mood (Chellappa et al., 2020). Furthermore, He et al. (2014) support our finding that stress mediated the association between shift work and depressed mood. Finally, analytical rumination did not mediate between shift work and depressed mood. To the best of our knowledge, such an association has yet to be established in the literature.

#### 4.2.3. Relationship between insomnia symptoms and depressed mood

Finally, we found a positive relationship between insomnia and depressed mood. For both SW and DW, experiencing insomnia was associated with more depressed mood. Furthermore, regression analyses showed that shift work, eveningness, stress, and depressed mood (but not analytical rumination), significantly explained insomnia symptoms; and likewise, depressed mood was significantly explained by all variables. Findings of a prospective study to assess predictors of insomnia among 800 nurses partially supported our findings, showing that depression increased the risk of insomnia, but insomnia did not increase the risk of depression (Vedaa et al., 2016). Another longitudinal study showed that insomnia served as a mediating factor between neuroticism and depression among approximately 2,000 nurses (Sørengaard et al., 2019). Given the cross-sectional design of the present study, we are not able to assume causality.

### 4.3. Strengths and limitations of the study

Our study has several strengths. This is a pioneering survey in assessing key phenomena—insomnia and depressed mood—that affect women in general and hospital female nurses in particular and in analyzing their occurrence rates separately and jointly for SW hospital nurses compared with DW nurses, who work only in the mornings. This assessment is important because of the mental burden that insomnia and depressed mood poses for nurses' life and work. Furthermore, we mapped the risk factors for the two phenomena and built an integrated conceptual psychobiological model through which we examined the connections between the risk factors from an adaptive-developmental point of view. We obtained a large sample of hospital nurses in Israel and performed the data collection anonymously thus minimizing potential bias.

This study has several limitations. First, it was conducted only among female nurses, albeit intentionally, since sleep disorders and emotional disorders are more common in women, and most hospital nurses in Israel are women (Israel Ministry of Health, 2018). However, results cannot be generalized to male hospital nurses. In addition, it is likely that there is sampling bias that limits generalization; so that nurses with moderate levels of insomnia and/or depressed mood tended to be more engaged in the survey, whereas those with most severe insomnia/worst mood, or conversely those with none of these conditions may have tended to decline from participation. This source of potential bias is supported by overall low rate of recruitment and the low response rate, both of which may be due to high workload, lack of time, and/or the sensitive topic of the study, especially depressed mood.

### 4.4. Recommendations for further research

To deepen the understanding of the mechanisms that contribute to the development of insomnia and depressed mood among the nursing population, we recommend studies that include objective sleep measurements (actigraphy) that include sleep quantity as well as quality, biological markers related to the development of insomnia, stress, depressed mood, and adaptation to shift work. Considering the findings of the reviewed studies, it is important to conduct longitudinal studies to identify causality and the possible contribution of additional personality factors to the relationship between insomnia and depressed mood through exposure to risk factors over time. Specifically, longitudinal studies can show whether analytical rumination as an adaptive mechanism, along with other coping mechanisms, is related to shift adjustment over time.

## Data availability statement

The raw data supporting the conclusions of this article will be made available by the authors, without undue reservation.

## Ethics statement

The studies involving human participants were reviewed and approved by the institutional Helsinki Committee (Approval Number 0496-16-RMB), the Rambam Hospital nursing administration, and the University of Haifa's faculty ethics committee (Approval Number 177/18). Based on the ethics committees' approvals, a statement was made at the beginning of the survey, stating that completion of the questionnaire indicated participants' consent.

## Author contributions

KB and TS contributed to conception and design of the study. KB organized the database, performed the statistical analysis, wrote the drafts of the manuscript. All authors contributed to manuscript revision, read, and approved the submitted version.
